# Epigenetic silencing of JAM3 promotes esophageal cancer development by activating Wnt signaling

**DOI:** 10.1186/s13148-022-01388-3

**Published:** 2022-12-02

**Authors:** Weili Yang, Chao Guo, James G. Herman, Cheng Zhu, Honghui Lv, Xiaomo Su, Lirong Zhang, Meiying Zhang, Mingzhou Guo

**Affiliations:** 1 Department of Gastroenterology and Hepatology, Chinese PLA General Hospital, #28 Fuxing Road, Beijing, 100853 China; 2 Laboratory Animal Center, Chinese PLA General Hospital, #28 Fuxing Road, Beijing, 100853 China; 3 The Hillman Cancer Center, University of Pittsburgh Cancer Institute, Pittsburgh, PA 15213 USA; 4 Medical College of NanKai University, Tianjin, 300071 China; 5 Henan Key Laboratory for Esophageal Cancer Research, Zhengzhou University, 40 Daxue Road, Zhengzhou, 450052 Henan China

**Keywords:** DNA methylation, Esophageal cancer, JAM3, Wnt signaling

## Abstract

**Background:**

The role of JAM3 in different tumors is controversial. The epigenetic regulation and the mechanism of JAM3 remain to be elucidated in human esophageal cancer (EC).

**Methods:**

Eleven EC cell lines, 49 cases of esophageal intraepithelial neoplasia (EIN) and 760 cases of primary EC samples were employed. Methylation-specific polymerase chain reaction, immunohistochemistry, MTT, western blot and xenograft mouse models were applied in this study.

**Results:**

The inverse association between RNA expression and promoter region methylation of JAM3 was found by analyzing 185 cases of EC samples extracted from the TCGA database (*p* < 0.05). JAM3 was highly expressed in KYSE450, KYSE520, TE1 and YES2 cells, low level expressed in KYSE70 cells and unexpressed in KYSE30, KYSE150, KYSE410, KYSE510, TE13 and BIC1 cells. JAM3 was unmethylated in KYSE450, KYSE520, TE1 and YES2 cells, partial methylated in KYSE70 cells and completely methylated in KYSE30, KYSE150, KYSE410, KYSE510, TE13 and BIC1 cells. The expression of JAM3 is correlated with methylation status. The levels of JAM3 were unchanged in KYSE450, KYSE520, TE1 and YES2 cells, increased in KYSE70 cells and restored expression in KYSE30, KYSE150, KYSE410, KYSE510, TE13 and BIC1 cells after 5-aza-2′-deoxycytidine treatment, suggesting that the expression of JAM3 is regulated by promoter region methylation. JAM3 was methylated in 26.5% (13/49) of EIN and 51.1% (388/760) of primary EC, and methylation of JAM3 was associated significantly with tumor differentiation and family history (all *p* < 0.05). Methylation of JAM3 is an independent prognostic factor of poor 5-year overall survival (*p* < 0.05). JAM3 suppresses cell proliferation, colony formation, migration and invasion and induces G1/S arrest and apoptosis in EC. Further study demonstrated that JAM3 suppressed EC cells and xenograft tumor growth by inhibiting Wnt/β-catenin signaling.

**Conclusion:**

JAM3 is frequently methylated in human EC, and the expression of JAM3 is regulated by promoter region methylation. JAM3 methylation is an early detection and prognostic marker of EC. JAM3 suppresses EC growth both in vitro and in vivo by inhibiting Wnt signaling.

## Background

Esophageal cancer (EC) is the seventh most common cancer and the sixth most common cause of cancer-related death worldwide. The five-year survival is below 20%, without changes in the past 15 years. Esophageal squamous cell carcinoma (ESCC) and adenocarcinoma (AC) are two major cell types, and ESCC is composed of 90% of EC. Eastern Asia exhibits the highest regional incidence, especially in the northern of China [1–3]. Alcohol and smoking are two major risk factors. Aberrant genetic and epigenetic changes play important roles in esophageal carcinogenesis and development, and epigenetic changes are mainly caused by environmental factors [4, 5]. So far, very limited driver mutations were found in EC, lacking hotspot mutations [6]. Dysregulation of cancer-related signaling pathways were found frequently by aberrant epigenetic changes [4, 7].

Junctional adhesion molecule (JAM) belongs to the immunoglobulin-like (Ig-like) superfamily of adhesion molecules [8]. Human JAM1 was cloned by comparing the homologue sequence of human EST bank with mouse JAM [9]. JAM2 was isolated and identified by the RNA differential display technique, and further sequence comparisons of JAM1 and JAM2 with EST databases identified another member of this new molecular family, JAM3 [10–12]. The JAM family shows both distinct and overlapping patterns of tissue expression, and all three JAM members contain consensus sequence, the PDZ binding motif. PDZ proteins play multifaceted roles in human disease by regulating protein trafficking, signal transduction, cell–cell junctions, cell polarity and adhesion [13]. The role and the mechanism of JAM3 are different in various cancers. JAM3 promotes renal cancer cell migration and inhibits its apoptosis [14]. By activating Wnt signaling, JAM3 plays an important role in the maintenance of leukemia-initiating cells stemness [15]. JAM3 serves as a tumor suppressor in colorectal cancer, and methylation of JAM3 is associated with cervical intraepithelial neoplasia malignant transformation [16, 17]. The role and the mechanism of JAM3 in esophageal cancer development remains to be elucidated.

## Results

### The expression of JAM3 is regulated by promoter region methylation in EC

**Fig. 1:**
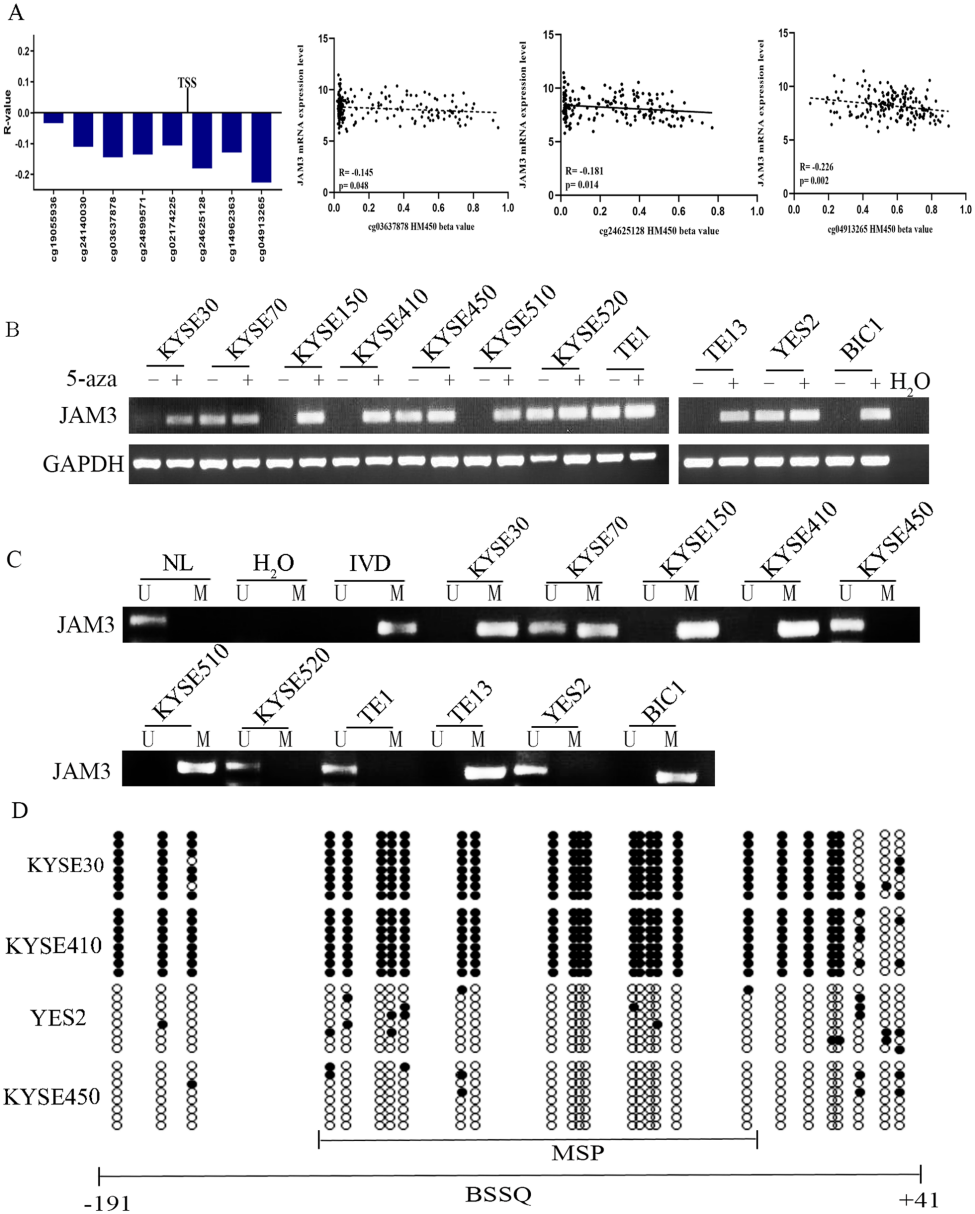
The expression and methylation status of JAM3 in esophageal cancer. The association of JAM3 expression and methylation around TSS site. Scatter plots show reduced JAM3 expression is associated with methylation status of cg03637878, cg24625128 and cg04913256. **b** Semiquantitative RT-PCR shows the expression of JAM3 in EC cells. KYSE30, KYSE70, KYSE150, KYSE410, KYSE450, KYSE510, KYSE520, TE1, TE13, YES2 and BIC1 are EC cells. 5-aza: 5-aza-2′-deoxycytidine; GAPDH: internal control of RT-PCR; H_2_O: double distilled water; (−): absence of 5-aza; (+): presence of 5-aza. **c** MSP results of JAM3 in EC cells. U: unmethylated alleles; M: methylated alleles; IVD: in vitro methylated DNA, serves as methylation control; NL: normal lymphocytes DNA, serves as unmethylation control; and H_2_O: double distilled water. **d** BSSQ results of JAM3 in KYSE30, KYSE410, YES2 and KYSE450 cells. The size of unmethylated MSP products was 137 bp, and bisulfite sequencing was focused on a 271 bp region around the JAM3 transcription start site (− 191 to + 41 bp). Filled circles: methylated CpG sites; open circles: unmethylated CpG sites; and TSS: transcription start site

TCGA database was employed to evaluate whether the expression of JAM3 is regulated by promoter region methylation in EC. RNA expression and methylation data of JAM3 were extracted from 185 cases of esophageal cancer samples in the TCGA database (http://xena.ucsc.edu/). As shown in Fig. 1a, inverse association was found between JAM3 expression and methylation in the promoter region around the transcript start site (TSS, cg03637878, cg24625128, cg04913265, all *p* < 0.05). The results hint that the expression of JAM3 is regulated by promoter region methylation in primary EC.

**Fig. 2:**
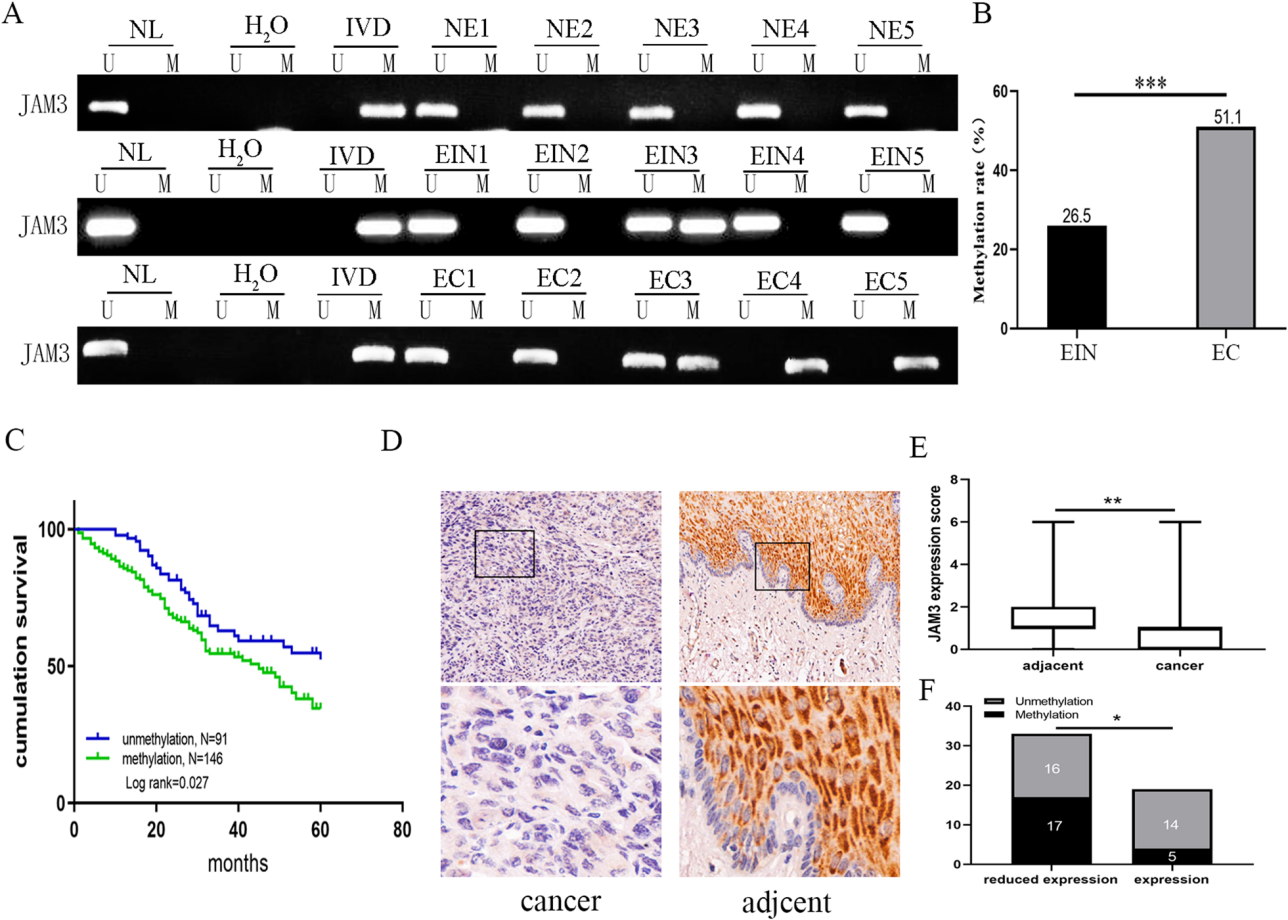
The methylation and expression status of JAM3 in EC samples. **a** Representative MSP results of JAM3 in normal esophageal mucosa and primary EC samples. NE: normal esophageal mucosa; EIN: esophageal intraepithelial neoplasia; EC: esophageal cancer samples. IVD: in vitro methylated DNA, serves as methylation control; NL: normal peripheral lymphocytes DNA, serves as unmethylation control; and H_2_O: double distilled water. **b** The frequency of JAM3 methylation in EIN and EC. *** *p* < 0.001. **c** The association of JAM3 methylation and OS of EC. **d** JAM3 staining in EC and adjacent tissue samples (top: 200 × ; bottom: 400 ×). **e** Box plots for JAM3 expression; horizontal lines represent the median score; the bottom and top of the boxes represent the 25th and 75th percentiles, respectively; vertical bars represent expression levels. **p < 0.01. **f** Bar diagram: the levels of JAM3 expression and methylation status. *p < 0.05

Further studies were performed to validate the epigenetic regulation of JAM3. The expression of JAM3 was detected by semiquantitative RT-PCR in human esophageal cancer cells. As shown in Fig. 1b, JAM3 was highly expressed in KYSE450, KYSE520, TE1 and YES2 cells, and low level expressed in KYSE70 cells, while it was unexpressed in KYSE30, KYSE150, KYSE410, KYSE510, TE13 and BIC1 cells. Unmethylation of JAM3 was detected in KYSE450, KYSE520, TE1 and YES2 cells, and partial methylation was found in KYSE70 cells, while it was completely methylated in KYSE30, KYSE150, KYSE410, KYSE510, TE13 and BIC1 cells (Fig. 1c). The methylation status is correlated with loss of/reduced expression of JAM3 in esophageal cancer cells. To further validate the expression of JAM3 is regulated by promoter region methylation, esophageal cancer cells was treated with 5-aza-2′-deoxycytidine (5-aza), an inhibitor of DNA methyltransferases. The levels of JAM3 were unchanged in KYSE450, KYSE520, TE1 and YES2 cells, increased in KYSE70 cells and re-expressed in KYSE30, KYSE150, KYSE410, KYSE510, TE13 and BIC1 cells (Fig. 1b). These results suggested that the expression of JAM3 is regulated by promoter methylation in EC cells. To rule out of the possibility of tissue/cell type-specific methylation, 5 cases of normal esophageal mucosa from non-cancerous patients were detected by MSP, and no methylation was found in these samples (Fig. 2a). For further validating the efficiency of the MSP primers, bisulfite sequencing was employed. Dense methylation was found in the promoter region of JAM3 in KYSE30 and KYSE410 cells, and unmethylation was found in YES2 and KYSE450 cells (Fig. 1d). The results were consistent with MSP detection in the cells, validating the efficiency of MSP primers.

### JAM3 is frequently methylated in human primary esophageal cancer

MSP technique was applied to detect the methylation status of JAM3. JAM3 was methylated in 26.5% (13/49) of esophageal intraepithelial neoplasia (EIN) and 51.1% (388/760) of primary EC. The frequency of JAM3 methylation was increased in a progression tendency during esophageal carcinogenesis (*p* < 0.001, Fig. 2a, b). Methylation of JAM3 was associated significantly with tumor differentiation and family history (all *p* < 0.05), but no association was found between JAM3 methylation and gender, age, tumor size, TNM stage, lymph node metastasis, alcohol consumption and smoking (all *p* > 0.05, Table 1).

**Table 1:** The association of JAM3 methylation and clinical factors in human EC

Clinical parameter	No	Methylation status		*p* value
		Methylated	Unmethylated	
	760	n = 388 (51.1%)	n = 372 (48.9%)	
*Gender*				
Male	528	272	256	0.7004
Female	232	116	116	
*Age*				
**< **60	196	109	87	0.1382
**≥ **60	564	279	285	
*Tumor size*				
**< **4 cm	247	132	115	0.3607
**≥ **4 cm	513	256	257	
*Differentiation*				
Moderately/Well	583	285	298	0.0300*
Poorly	177	103	74	
*TNM stage*				
I/II	383	198	185	0.7202
III/IV	377	190	187	
*Lymph node metastasis*				
Negative	367	187	180	0.9579
Positive	393	201	192	
*Smoking*				
No	422	221	201	0.4170
Yes	338	167	171	
*Drinking*				
No	547	284	263	
Yes	213	104	109	0.4436
*Family history*				
No	524	255	269	0.0497*
Yes	236	133	103	

*P values are obtained from χ^2^ test, significant difference, *p* < 0.05*

For 237 cases of EC with available data for overall 5-year survival, the association of JAM3 methylation and survival time was analyzed by a Cox multivariable proportional hazards and Kaplan–Meier model. As shown in Fig. 2c and Table 2, methylation of JAM3 is significantly associated with poor 5-year OS (*p* < 0.05) and is an independent poor prognostic factor for 5-year OS (*p* < 0.05, Table 2).

**Table 2:** COX multivariate analysis of JAM3 methylation status with 5-year survival in EC patients (n = 237)

Clinical parameter	HR (95%CI)	*p* value
Gender (male vs. female)	1.336 (0.768–2.326)	0.305
Age (≥ 60 vs. < 60 years)	1.462 (0.932–2.293)	0.099
Tumor size (≥ 4 vs. < 4 cm)	1.143 (0.756–1.727)	0.527
Differentiation (low vs. high or middle)	1.354 (0.891–2.057)	0.156
TNM stage (3–4 vs. 1–2)	1.276 (0.504–3.231)	0.607
Lymph node metastasis (positive vs. negative)	1.572 (0.597–4.141)	0.360
JAM3 (methylation vs. unmethylation)	1.545 (1.019–2.343)	0.041*
Smoking (yes vs. no)	0.858 (0.523–1.407)	0.544
Drinking (yes vs. no)	1.381 (0.869–2.195)	0.172
family history (yes vs. no)	0.785 (0.520–1.184)	0.248

*HR* hazard ratio, *CI* confidence interval

*p < 0.05

The expression of JAM3 is examined by immunohistochemistry (IHC) in 52 cases of available matched esophageal cancer and adjacent tissue paraffin samples. As shown in Fig. 2d, e, JAM3 is highly expressed in adjacent tissue samples and reduced in primary cancer samples (*p* < 0.01), and JAM3 is mainly located in the cell membrane and cytoplasm. The reduced expression of JAM3 is associated with the promoter region methylation (*p* < 0.05, Fig. 2f). The results further suggest that the expression of JAM3 is regulated by promoter region methylation in primary EC.

### JAM3 suppresses esophageal cancer cell proliferation

**Fig. 3:**
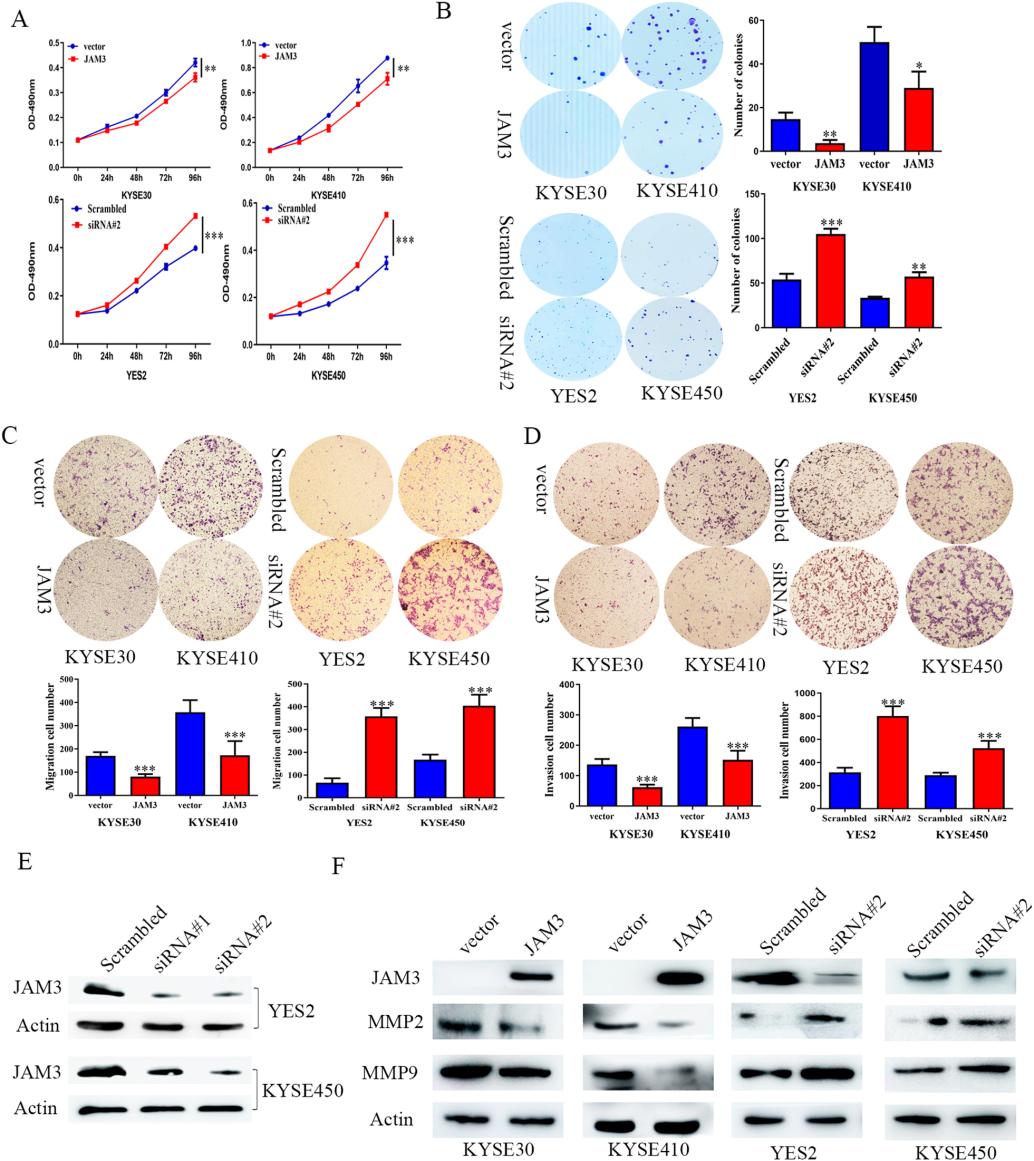
The effect of JAM3 on cell proliferation, invasion and migration. **a** Growth curves represent the effects of JAM3 in EC cells. **b** Colony formation results show that the colony number was reduced by re-expression of JAM3 in KYSE30 and KYSE410 cells, and increased by knockdown of JAM3 in YES2 and KYSE450 cells. The average number of tumor clones is represented by the bar diagram. **c** Cell migration experiment in KYSE30 and KYSE410 cells before and after re-expression of JAM3, and in YES2 and KYSE450 cells before and after knockdown of JAM3. The average number of migration cells is presented by a bar diagram. **d** Cell invasion experiment in KYSE30 and KYSE410 cells before and after re-expression of JAM3, and in YES2 and KYSE450 cells before and after knockdown of JAM3. The average number of invasion cells is presented by a bar diagram. Each experiment was repeated in triplicate. *p < 0.05. **p < 0.01. ***p < 0.001. **e** Western blots show the effects of knockdown of JAM3 by different siRNA. Scrambled: siRNA negative control; siRNA#1 and siRNA#2: siRNA for JAM3. **f** The levels of JAM3, MMP2 and MMP9 were detected by western blot. Actin: internal control. Scrambled: siRNA negative control; siRNA#2: siRNA targeting JAM3

By detecting the efficiency of siRNA for JAM3, siRNA#2 was found more effective than siRNA#1, and siRNA#2 was applied to further study (Fig. 3e). To evaluate the effect of JAM3 on cell viability, MTT assay was employed. The OD values were 0.421 ± 0.016 versus 0.361 ± 0.017 and 0.878 ± 0.008 versus 0.711 ± 0.049 before and after restoration of JAM3 expression in KYSE30 cells and KYSE410 cells, respectively. The OD values were reduced significantly in KYSE30 cells and KYSE410 cells after restoration of JAM3 expression (both *p* < 0.01), suggesting that JAM3 suppresses EC cell proliferation. The effect of JAM3 on cell growth was further validated by knocking down JAM3 in JAM3 highly expressed YES2 and KYSE450 cells. The OD values were 0.398 ± 0.002 versus 0.532 ± 0.011, and 0.347 ± 0.026 versus 0.551 ± 0.006 before and after knockdown of JAM3 in YES2 and KYSE450 cells, respectively (Fig. 3a). The OD values were increased significantly after knockdown of JAM3 in YES2 and KYSE450 cells (both *p* < 0.001), further suggesting that JAM3 suppresses EC cell proliferation.

To evaluate the effects of JAM3 on clonogenicity in EC cells, colony formation assay was performed. The clone number was 14.7 ± 3.1 versus 3.7 ± 1.5 in KYSE30 cells (*p* < 0.01) and 50.0 ± 7.0 versus 29.0 ± 7.5 in KYSE410 cells (*p* < 0.05) before and after restoration of JAM3 expression, respectively (Fig. 3b). The clone number was reduced significantly after re-expression of JAM3 in KYSE30 and KYSE410 cells. To further validate the effect of JAM3 on clonogenicity, siRNA knockdown technique was employed. The clone number was 54.0 ± 6.6 versus 105.0 ± 6.1 and 33.7 ± 1.1 versus 57.3 ± 5.0 before and after knockdown of JAM3 in YES2 and KYSE450 cells, respectively (Fig. 3b). The clone number was increased significantly after knockdown of JAM3 in YES2 and KYSE450 cells (*p* < 0.001, *p* < 0.01). These results suggest that JAM3 inhibits cell proliferation in EC.

### JAM3 suppresses cell migration and invasion in esophageal cancer cells

Transwell assay was employed to evaluate the effects of JAM3 on cell migration and invasion. The number of migratory cells was 170.3 ± 15.9 versus 80.8 ± 11.1 (*p* < 0.001) and 337.7 ± 27.5 versus 133.5 ± 23.0 (*p* < 0.01) before and after restoration of JAM3 expression in KYSE30 and KYSE410 cells, respectively. The number of migratory cells was 66.2 ± 19.7 versus 357.7 ± 37.1 (*p* < 0.001) and 181.2 ± 9.5 versus 386.2 ± 13.8 (*p* < 0.001) before and after knockdown of JAM3 in YES2 and KYSE450 cells, respectively (Fig. 3c). The number of migratory cells was significantly reduced after restoration of JAM3 expression in KYSE30 and KYSE410 cells and increased significantly after knockdown of JAM3 expression in YES2 and KYSE450 cells, suggesting that JAM3 suppresses esophageal cancer cell migration. The number of invasive cells was 136.8 ± 18.0 versus 62.1 ± 8.4 (*p* < 0.001) in KYSE30 cells and 272.0 ± 54.9 versus 144.6 ± 22.3 (*p* < 0.001) in KYSE410 cells before and after restoration of JAM3 expression, respectively. The number of invasive cells was reduced significantly after restoration of JAM3 expression in KYSE30 and KYSE410 cells (Fig. 3d). The number of invasive cells was 315.7 ± 38.6 versus 801.8 ± 84.2 (*p* < 0.001) and 290.2 ± 21.5 versus 524.0 ± 63.4 (*p* < 0.001) before and after knockdown of JAM3 in YES2 and KYSE450 cells, respectively (Fig. 3d). Above results suggest that JAM3 suppresses EC cell invasion.

The roles of JAM3 in cell migration and invasion were further validated by analyzing the expression levels of MMP2 and MMP9 with western blot. As shown in Fig. 3f, the levels of MMP2 and MMP9 expression were reduced after restoration of JAM3 in KYSE30 and KYSE410 cells and increased after knockdown of JAM3 in YES2 and KYSE450 cells. These results further demonstrated that JAM3 suppresses EC cell migration and invasion.

### JAM3 induces G1/S arrest in esophageal cancer cells

**Fig. 4:**
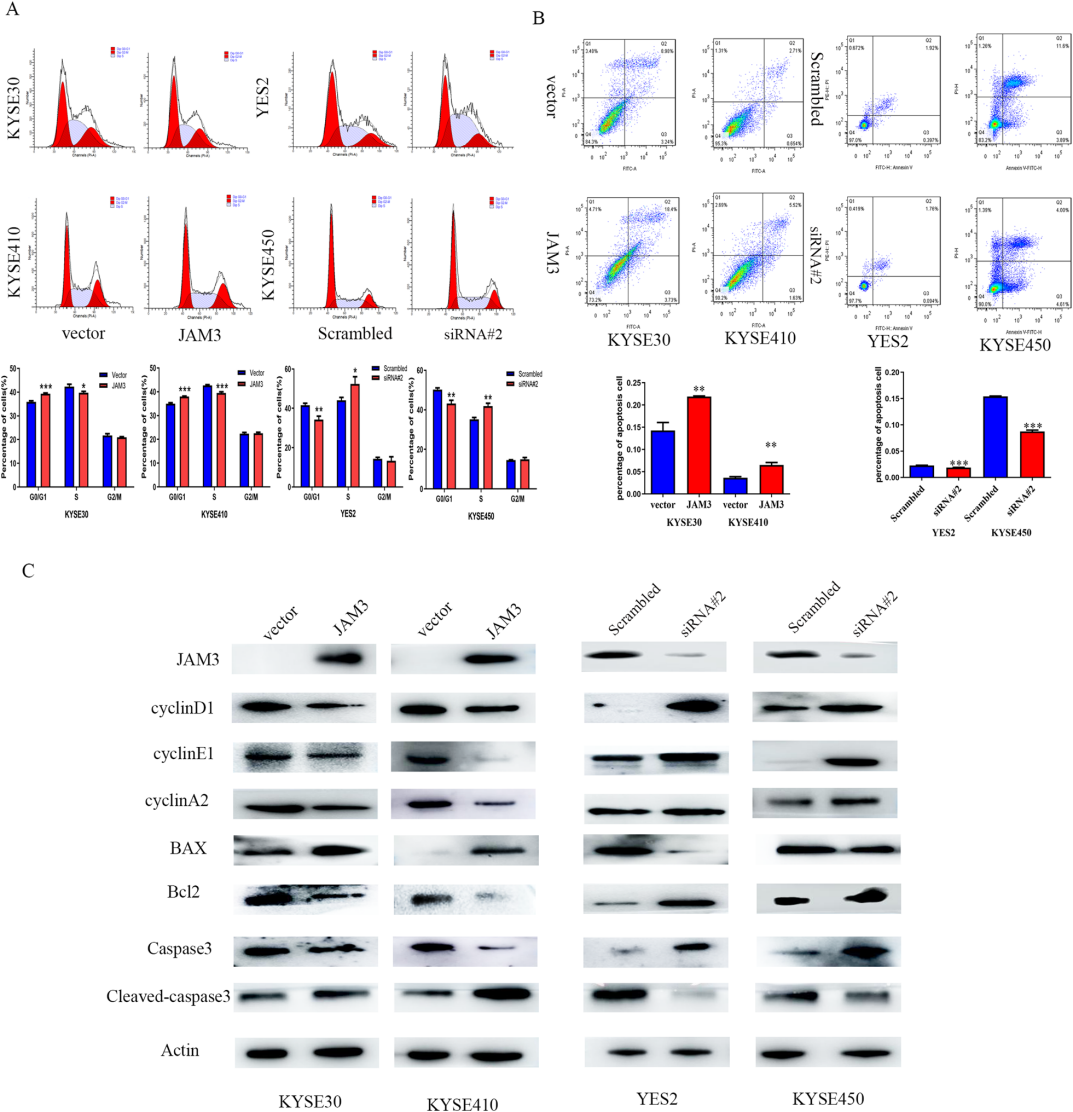
The effect of JAM3 on cell cycle and apoptosis. **a** The effect of JAM3 on cell phase in EC cells. The bar diagram represents the percentage. **b** The effect of JAM3 on apoptosis in EC cells. Each experiment was repeated in triplicate. *p < 0.05. **p < 0.01. ***p < 0.001. **c** Western blot shows the levels of JAM3, cyclinD1, cyclin E1, cyclin A2, BAX, Bcl2, caspase3 and cleaved-caspase3. Actin: internal control. Scrambled: siRNA negative control; siRNA#2: siRNA targeting JAM3

The role of JAM3 in cell cycle was analyzed by flow cytometry. The cell phase distributions were 35.93 ± 0.40% versus 39.32 ± 0.34% in G1 phase (*p* < 0.001); 42.35 ± 0.96% versus 39.74 ± 0.57% in S phase (*p* < 0.05); and 21.72 ± 0.75% versus 20.93 ± 0.31% in G2/M phase before and after re-expression of JAM3 in KYSE30 cells, respectively. The cell phase distributions were 34.98 ± 0.44% versus 37.97 ± 0.22% in G1 phase (*p* < 0.001); 42.66 ± 0.31% versus 39.47 ± 0.50% in S phase (*p* < 0.001); and 22.36 ± 0.52% versus 22.56 ± 0.41% in G2/M phase before and after re-expression of JAM3 in KYSE410 cells, respectively. The number of G1 phase cells is increased significantly, and S phase cell is reduced significantly after re-expression of JAM3 in KYSE30 and KYSE410 cells (Fig. 4a).

To further validate the role of JAM3 in cell cycle, the cell phase distributions were also analyzed in YES2 and KYSE450 cells before and after knockdown of JAM3. The cell phase distributions were 41.59 ± 0.96% versus 34.24 ± 1.77% in G1 phase (*p* < 0.01); 44.06 ± 1.44% versus 52.41 ± 3.66% in S phase (*p* < 0.05); and 14.35 ± 0.72% versus 13.35 ± 2.01% in G2/M phase in YES2 cells before and after knockdown of JAM3. The cell phase distributions were 50.24 ± 0.82% versus 43.16 ± 1.67% in G1 phase (*p* < 0.01); 35.17 ± 0.97% versus 41.91 ± 1.40% in S phase (*p* < 0.01); and 14.58 ± 0.16% versus 14.93 ± 0.94% in G2/M phase in KYSE450 cells, before and after knockdown of JAM3. The number of G1 phase cells is reduced significantly, and S phase cells are increased after knockdown of JAM3 in YES2 and KYSE450 cells (Fig. 4a, all *p* < 0.05). Above results demonstrated that JAM3 induced G1/S arrest in EC cells.

To further validate the effect of JAM3 on cell cycle, the levels of cell cycle-related proteins were detected by western blot. As shown in Fig. 4c, the levels of cyclinA2, cyclinD1 and cyclinE1 were decreased after re-expression of JAM3 in KYSE30 and KYSE410 cells. The levels of cyclinA2, cyclinD1 and cyclinE1 were increased after knockdown of JAM3 in YES2 and KYSE450 cells (Fig. 4c). These results further validated the effect of JAM3 on induction of G1/S phase arrest in EC cells.

### JAM3 induces apoptosis in EC cells

Flow cytometry technique was used to analyze cell apoptosis. The percentage of apoptotic cells was 14.9 ± 1.9% versus 21.7 ± 0.5%, and 3.63 ± 0.24% versus 6.5 ± 0.57% in JAM3 unexpressed and re-expressed KYSE30 and KYSE410 cells, respectively. The ratio of apoptotic cells was increased significantly after re-expression of JAM3 in KYSE30 and KYSE410 cells (both *p* < 0.01, Fig. 4b). The percentage of apoptotic cells was 2.22 ± 0.08% versus 1.96 ± 0.08%, and 15.31 ± 0.15% versus 8.9 ± 0.29% in YES2 and KYSE450 cells before and after knockdown of JAM3. The ratio of apoptotic cells was reduced significantly after knockdown of JAM3 in YES2 and KYSE450 cells (both *p* < 0.001, Fig. 4b). The apoptosis-related proteins were detected by western blot. As shown in Fig. 4c, the levels of Caspase3 and Bcl2 were decreased and BAX and Cleaved-caspase3 were increased after restoration of JAM3 expression in KYSE30 and KYSE410 cells, and the levels of Caspase3 and Bcl2 were increased and BAX and Cleaved-caspase3 were decreased after knockdown of JAM3 in YES2 and KYSE450 cells. These results further suggest that JAM3 induces apoptosis in EC cells.

### JAM3 inhibits Wnt/β-Catenin signaling in esophageal cancer

**Fig. 5:**
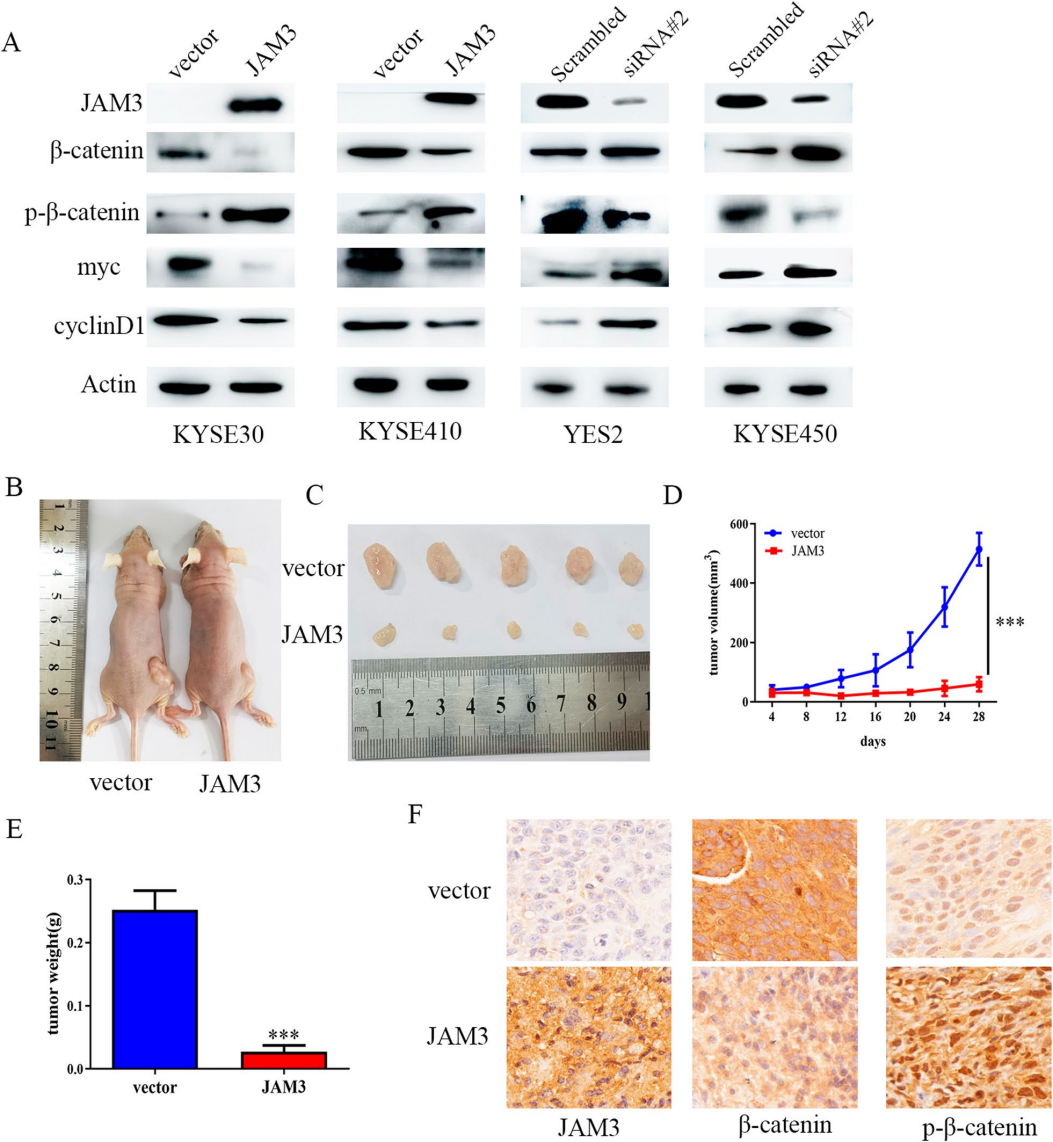
The effect of JAM3 on Wnt/β-catenin signaling in EC. **a** Western blot shows the levels of JAM3, β-catenin, p-β-catenin, Myc and cyclinD1 in EC cells. Actin: internal control. Scrambled: siRNA negative control; siRNA#2: siRNA targeting JAM3. **b, c** Representative tumors from JAM3 unexpressed and re-expressed KYSE410 cell xenografts in mice. **d** Growth curves of JAM3 unexpressed and re-expressed KYSE410 cell tumors. ***p < 0.001. **e** Tumor weight in JAM3 unexpressed and re-expressed KYSE410 cell xenografts. Bars represent the mean of tumor weight. ***p < 0.001. **f** Representative results of immunohistochemistry for JAM3, β-catenin and p-β-catenin in xenografts

JAM3 have been reported to be involved in Wnt signaling in leukemia initiation [15]. Western blot was employed to determine whether JAM3 is involved in Wnt signaling pathway in human esophageal cancer. The levels of β-catenin were reduced, and the levels of phospho-β-catenin were increased after re-expression of JAM3 in KYSE30 and KYSE410 cells. The levels of Myc and cyclinD1, the downstream genes of Wnt signaling, were reduced after re-expression of JAM3 in KYSE30 and KYSE410 cells (Fig. 5a). The results hint that JAM3 inhibits Wnt signaling in human EC. In JAM3 highly expressed YES2 and KYSE450 cells, the levels of β-catenin, Myc and cyclinD1 were increased and the levels of phospho-β-catenin were reduced after knockdown of JAM3 (Fig. 5a). The results suggested that JAM3 inhibits Wnt signaling in human EC.

### JAM3 suppresses EC cell xenografts growth by inhibiting Wnt signaling

To further validate the roles of JAM3 in esophageal cancer growth, EC cell xenograft mouse models were employed. As shown in Fig. 5b, c, the tumor volume was 514.83 ± 55.08mm^3^ and 59.93 ± 24.28mm^3^ in JAM3 unexpressed and re-expressed KYSE410 cells xenografts. The tumor volume was significantly smaller in JAM3 re-expressed KYSE410 cell xenografts than in JAM3 unexpressed KYSE410 cell xenografts (Fig. 5d, *p* < 0.001). The tumor weight was 0.252 ± 0.030 g and 0.027 ± 0.010 g in JAM3 unexpressed KYSE410 cell xenografts and in JAM3 re-expressed KYSE410 cell xenografts. The xenograft weight was reduced significantly after re-expression of JAM3 in KYSE410 cell compared to JAM3 unexpressed KYSE410 cell xenografts (Fig. 5e, *p* < 0.001). To evaluate the effects of JAM3 on Wnt signaling in vivo, the expression levels of JAM3, β-catenin and p-β-catenin were detected by IHC in xenografts. The levels of p-β-catenin were increased, and the levels of β-catenin were reduced in JAM3 re-expressing EC cell xenografts (Fig. 5f). These results suggest that JAM3 suppresses Wnt signaling in EC cell xenografts.

## Discussion

Even though JAM3 was reported to be involved in different cancers, its role in cancer initiation and development is still in controversial. JAM family members (JAM1, 2, 3) have a conserved PDZ binding motif [11]. Proteins containing PDZ domains are predominantly localized to the plasma membrane and are recruited to specialized sites of cell–cell contact. Many PDZ domains mediate protein–protein interactions by interacting with short consensus motifs found at the free carboxyl terminus of transmembrane proteins [10, 11]. JAM3 plays its role by binding intracellular components through its c-terminal PDZ binding motif [11]. PDZ proteins contribute to cytoskeletal dynamics for maintaining cell–cell junctions, cell polarity and cell migration, and their dysfunction can lead to multiple diseases [13]. JAM3 plays distinct roles in different cell types or diseases [14, 16, 18]. The mechanisms of EC development remain poorly understand. Thus, we explored the mechanism and the epigenetic regulation of JAM3 in EC. JAM3 was found methylated in 26.5% (13/49) of esophageal precancerous lesions and 51.1% (388/760) of primary EC, and the expression of JAM3 was regulated by promoter region methylation. Methylation of JAM3 was associated significantly with tumor differentiation and family history. Further analysis suggests that methylation of JAM3 is an independent prognostic factor for poor 5-year OS. These results suggest that JAM3 methylation may serve as an early detection and prognostic marker of EC. Germline gene mutations were regarded as the cause of family cancers [19, 20]. So far, no germline mutations were found in family EC patients in the high incidence area [6]. Environmental factors were regarded as major causes of aberrant epigenetic changes in human cancer [7, 21–23], explaining the phenomenon of JAM3 methylation with family history. Our previous study has found that some transcriptional factors were cancer type specific methylation, including CDX2 and APC genes [5, 24–26]. CDX2 is frequently methylated in human ESCC, but unmethylated in colorectal cancer and esophageal adenocarcinoma [25]. CDX2 was regarded as a tumor suppressor in ESCC and an oncogene in colorectal cancer [25, 27]. Cell fate-determining genes play important roles in development and cancer [28, 29]. The aberrant epigenetic changes of cell fate-determining genes may serve as “synthetic lethality” therapeutic targets [30, 31]. JAM3 plays conflicting roles in different cancers [14–16, 18]. In EC, JAM3 inhibits cell proliferation, migration and invasion and induces G1/S arrest and cell apoptosis. JAM3 suppresses KYSE410 cell xenograft tumor growth in vivo. Further study suggests that JAM3 suppresses EC growth by inhibiting Wnt signaling. In addition to JAM3, opposing roles in promoting and suppressing tumor growth have been reported for the same molecules in different cell type of cancers [25, 27, 32]. Contrasting roles in hepatic cellular carcinoma have also been observed for β-catenin, a chief effector in the Wnt pathway [33, 34]. Either aberrant gain or loss of function of the same molecule can lead to malignant growth, reinforcing the concept that homeostasis is critical for preventing tumorigenesis. It is challenging to complete understand the mechanisms of conflicting roles of the same molecule. The opposing roles in promoting and suppressing cancers are not restricted to intracellular signaling molecules. Microenvironment factors play important roles in signaling transduction, such as inflammation and paracrine cytokines [35–37]. One molecule may present multifaceted roles by interacting with distinct proteins in different signaling pathways [13]. Dissecting these underlying mechanisms may create novel therapeutic strategies. Treatment of BRCA1/2 mutational breast cancer with PARP inhibitor is a paradigm of “Synthetic lethality” therapy [29]. It is necessary to complete understand the network of signaling transduction in different cancers for innovation therapeutic strategies beyond “BRCAness” [31, 38]. Aberrant epigenetic changes occurred more frequently compared to driver gene mutations in cancer. It is possible to make breakthrough in precision medicine by better understanding epigenetic based “Synthetic lethality” therapeutic strategy. In future study, it will improve the treatment of EC by clarifying the regulation network of JAM3.

## Conclusions

JAM3 is frequently methylated in human esophageal cancer, and the expression of JAM3 is regulated by promoter region methylation. JAM3 methylation is an early detection and prognostic marker of EC. JAM3 suppresses EC growth both in vitro and in vivo by inhibiting Wnt signaling.

## Materials and methods

### Human tissue samples and cell lines

Human tissue samples were collected from the Chinese PLA General Hospital in Beijing, including a total of 760 cases of primary EC, 49 cases of EIN (11 cases of low grade EIN and 38 cases of high grade EIN) and 5 cases of normal esophageal mucosa from non-cancerous patients. The median age of the patients with cancer is 65 years old (range 39–87 y), and the ratio is 2.3/1 for men/women. All cancer samples were classified according to TNM staging (AJCC 2019), including 68 cases of stage I, 315 cases of stage II, 331 cases of stage III and 46 cases of stage IV. All samples were collected from patients without chemo-radiotherapy before surgery. Samples were collected under the guidelines approved by the institutional review board at the Chinese PLA General Hospital with written informed consent from patients.

Eleven EC cell lines (KYSE30, KYSE70, KYSE150, KYSE410, KYSE450, KYSE510, KYSE520, TE1, TE13, YES2 and BIC1) were included in this study. All EC cell lines were previously established from primary esophageal cancer and maintained in RPMI-1640 (Invitrogen, USA) supplemented with 10% fetal bovine serum and 1% penicillin/streptomycin solution (Sigma, USA). All cell lines were cultured at 37 °C in an atmosphere of 5% carbon dioxide.

### 5-Aza-2′-deoxycytidine treatment, RNA preparation and semiquantitative RT-PCR

EC cell lines were split to a low density (30% confluence) 12 h before treatment. Cells were treated with 5-aza-2′-deoxycytidine (5-aza, Sigma, USA) at a concentration of 2 μM. Growth medium conditioned with 5-aza at a concentration of 2 μM was exchanged every 24 h for 96 h. The total RNA was prepared by TRIzol reagent (Life Technology, USA). Agarose gel electrophoresis and spectrophotometric analysis were used to detect RNA quality and quantity. Total RNA (5 μg) was used to synthesize first strand cDNA according to the manufacturer’s instructions (Invitrogen, USA). The reaction mixture was diluted to 100 μl with water, and then 2.5 μl of diluted cDNA mixture was used for 25 μl PCR reaction. The PCR primer sequences for JAM3 were as follows: 5′-CTGGAATGTGACACGGAGAGAC-3′ (F) and 5′-CCTTCGGCACTCTACAGACAG-3′ (R). Semiquantitative reverse transcription PCR (RT‐PCR) was amplified for 35 cycles. GAPDH was amplified for 25 cycles as an internal control. GAPDH primer sequences were as follows: 5′-GACCACAGTCCATGCCATCAC-3′ (F), and 5′-GTCCACCACCCTGTTGCTGTA-3′ (R). The amplified PCR products were examined by 1.5% agarose gels.

### DNA extraction, bisulfite modification, methylation-specific PCR and bisulfite sequencing

Genomic DNA was prepared by the proteinase K method. The bisulfite treatment was performed as previously described [39]. Methylation-specific PCR (MSP) primers were designed according to genomic sequences around the TSS and synthesized (BGI, China) to detect unmethylated (U) and methylated (M) alleles. MSP primer sequences were as follows: 5′-TTATGGTGTCGGTTCGGTTGGGTTC-3′(MF) and 5′-AATTACTAAAAAACCAACGACAACGCG-3′(MR); 5′-TTATTATGGTGTTGGTTTGGTTGGGTTT-3′(UF) and 5′-AAAAATTACTAAAAAACCAACAACAACACA-3′(UR). The expected size of unmethylated and methylated products was 137 bp and 134 bp, respectively. Bisulfite-treated DNA was amplified using bisulfite sequencing (BSSQ) primers that included the MSP amplification region. The bisulfite sequencing primers were as follows: 5′-TTAAGTTTATTGAAAGAGAATTTATGTGT-3′(F) and 5′-ATCAAACAACCRAACRCAAAACCRAA-3′(R). Bisulfite sequencing was performed as previously described [40].

### Immunohistochemistry

Immunohistochemistry (IHC) was performed in primary EC samples and paired adjacent tissue samples. JAM3 antibody (Abcam, USA), phosphorylated β-catenin (p-β-catenin) antibody (ZENBIO, China) and β-catenin antibody (Cell Signaling Technology, USA) were diluted to 1:25, 1:30 and 1:500, respectively. For antigen retrieval, the slides were placed in citrate antigen-repairing solution and heated in a high-pressure cooker until steam arose. The slides were kept inside the cooker for 150 s and then cooled down at room temperature for 15 min. The intensity and the scope of staining were scored using the German semiquantitative scoring system as described previously [40, 41].

### Construction of JAM3 expression lentiviral vectors and screening of JAM3 expression cells

The human full-length JAM3 cDNA was cloned into the pCDH-CMV-MCS-EF1-Puro vector. The primers sequences were as follows: 5′-CCGGAATTCATGGCGCTGAGGCGGCCACCG-3′(F) and 5′-CGCGGATCCTCAGATCACAAACGATGACTTGTGTC-3′(R). The HEK-293 T cells were maintained in 90% DMEM (Invitrogen, USA) supplemented with 10% fetal bovine serum. JAM3 expressing lentiviral or empty vectors were transfected into HEK-293 T cells (5 × 10^6^ per 100 mm dish) using the ViraPower™ lentiviral expression systems (Invitrogen, USA). Viral supernatant was collected and filtered after 48 h. Lentivirus was added to the growing medium of KYSE30 and KYSE410 cells, and JAM3 stably expressed cells were selected using puromycin (MCE, USA) at a concentration of 2.5 μg/mL for 3 days.

### MTT and colony formation assays

Cells were seeded into 96-well plates at a density of 1 × 10^3^ cells/well, and the cell viability was measured by the MTT assay (KeyGEN Biotech, China) at 0, 24, 48, 72 and 96 h. Absorbance was measured on a microplate reader (Thermo Multiskan MK3, USA) at a wavelength of 490 nm. The results were plotted as means ± SD.

For colony formation assay, cells were seeded in 6-well plates at a density of 300 cells/well growing for 14 days. Cells were fixed with 75% ethanol for 30 min and stained with 0.2% crystal violet (Beyotime, China), and then, the number of clones was counted. Each experiment was repeated for three times.

### Cell cycle and apoptosis analysis

For cell cycle analysis, all cells were starved for 12 h for synchronization, including KYSE30 and KYSE410 cells with and without expression of JAM3, and YES2 and KYSE450 cells before and after knockdown of JAM3. Then, the cells were re-stimulated with 10% FBS for 48 h. Cells were fixed with 70% ethanol and stained with propidium following the protocol of the Cell Cycle Detection Kit (KeyGEN Biotech, China). The cells were then sorted by a FACS Caliber (BD Biosciences, CA), and cell phase distribution was analyzed by the Modifit software (Verity Software House, USA). Each experiment was repeated for three times.

For apoptosis analysis, the cells were prepared following the manufacturer’s instruction of the Annexin V-FITC/PI Apoptosis Detection Kit (KeyGen Biotech, China) and analyzed by a FACScan Flow Cytometer (Becton–Dickinson Biosciences, MA). Each experiment was repeated for three times.

### Transwell assay

For migration study, cells (1 × 10^5^) were suspended in 200 μl serum-free RPMI-1640 media and added to the upper chamber of an 8.0 μm pore size transwell apparatus for 28 h (COSTAR, Corning Incorporated, USA). Then, the cells, which migrated to the surface of lower chamber membranes, were stained with crystal violet and counted in three independent high-power fields (× 200). Each experiment was repeated for three times.

For invasion study, cells (2 × 10^5^) were seeded into the upper chamber of a transwell apparatus coated with Matrigel (BD Biosciences, CA) and incubated for 36 h. Then, cells invaded to the membrane surface of lower chamber were stained with crystal violet and counted in three independent high-power fields (× 200). Each experiment was repeated for three times.

### SiRNA knockdown technique

The sequences for two sets of JAM3 targeting siRNA and one set of RNAi scrambled control duplex are as follows: siRNA#1 duplex for JAM3 (sense: 5′-GGUUCUUGUAACUUUCUCCAUCCUGAU-3′; antisense: 5′-AUCAGGAUGGAGAAAGUUACAAGAACC-3′); siRNA#2 duplex for JAM3 (sense: 5′-CAGGAUGGAGAAAGUUACAAGAACC-3′; antisense: 5′-GGUUCUUGUAACUUUCUCCAUCCUG-3′); and RNAi scrambled control duplex (sense: 5′-UUCUCCGAACGUGUCACGUTT-3′; antisense: 5′-ACGUGACACGUUCGGAGAATT-3′). RNAi oligonucleotide and RNAi scrambled control duplex (JTS scientific, China) were transfected into JAM3 highly expressed cells. SiRNA#2 was found more effective than siRNA#1, and siRNA#2 was applied for subsequent experiments.

### Protein preparation and western blot

Proteins were prepared, and western blots were performed as described previously [41]. Antibodies were diluted according to the manufacturer’s instructions, including JAM3 (Abcam, USA), Caspase3/Cleaved-caspase3 (Proteintech, USA), MMP2 (Proteintech, USA), MMP9 (Proteintech, USA), cyclinD1 (Proteintech, USA), cyclin A (Proteintech, USA), cyclin E1 (Proteintech, USA), Myc (Proteintech, USA), β-catenin (Proteintech, USA), p-β-catenin (ZENBIO, China) and Actin (Proteintech, USA).

### The effects of JAM3 on KYSE410 cell xenograft

JAM3 stably expressed and unexpressed KYSE410 cells (4 × 10^6^ cells in 0.15 ml phosphate-buffered saline) were subcutaneously injected into the dorsal right side of 4-week-old BABL/c nude mice. The tumor volume was measured every 3 days for each time for a total of 28 days, starting at the 4th day after implantation. Tumor volume was calculated according to the formula: V = L × W^2^/2, where V represents volume (mm^3^), L represents the biggest diameter (mm), and W represents the smallest diameter (mm). All procedures were approved by the Animal Ethics Committee of the Chinese PLA General Hospital.

### Statistical analysis

SPSS 22.0 software (IBM, NY, USA) was applied using χ^2^ test for independent dichotomous variables. All data were presented as means ± standard deviation (SD) for at least three independent experiments and analyzed using the Student’s t test. Kaplan–Meier plots and the log-rank test were used to estimate the effect of 2 experimental groups in overall survival (OS). The association of risk factors (gender, age, tumor size, tumor differentiation, TNM stage, lymph node metastasis, JAM3 methylation, smoking, alcohol consumption and family history) with the five-year OS was assessed by multivariate Cox proportional hazards regression models. *p* < 0.05 was considered as statistically significant.

## Data Availability

Based on a reasonable request, the data from the current research analysis can be obtained from the corresponding author.

## Abbreviations

5-aza: 5-Aza-2′-deoxycytidine
EC: Esophageal cancer
JAM3: Junctional adhesion molecule 3
GAPDH: Glyceraldehyde-3-phosphate dehydrogenase
IHC: Immunohistochemistry
IVD: In vitro-methylated DNA
NL: Normal lymphocyte DNA
MSP: Methylation-specific PCR
BSSQ: Bisulfite sequencing
TSS: Transcription start site
TCGA: The Cancer Genome Atlas
